# A Review of Respiratory Infection Caused by the Human Metapneumovirus: Occurrence, Epidemiology, Pathogenesis, Public Health Outcomes, and Intervention Strategies

**DOI:** 10.1002/hsr2.71962

**Published:** 2026-03-23

**Authors:** Sylvester Chibueze Izah, Matthew Chidozie Ogwu

**Affiliations:** ^1^ Department of Community Medicine Faculty of Clinical Sciences Bayelsa Medical University Yenagoa Bayelsa State Nigeria; ^2^ Goodnight Family Department of Sustainable Development Appalachian State University Boone North Carolina USA

**Keywords:** antiviral therapies, coinfection, epidemiology, pathogenesis, public health outcomes, respiratory infections, surveillance systems

## Abstract

**Background and Aims:**

Human metapneumovirus (hMPV) is a leading cause of acute respiratory tract infections, particularly in children, older adults, and individuals with immunocompromising conditions. Despite increasing global recognition of its clinical relevance, a comprehensive understanding of hMPV's epidemiology, pathogenesis, and intervention strategies remains limited. This review aims to summarize recent advances in hMPV research, highlight knowledge gaps, and outline future directions for research and public health preparedness.

**Methods:**

We conducted a structured narrative review of peer‐reviewed literature on hMPV published between 2001 and 2025, with emphasis on recent studies (2015–2024). Databases searched included Google Scholar, PubMed, Scopus, and Web of Science. Articles were selected based on relevance to epidemiological patterns, clinical features, diagnostic approaches, molecular virology, immunopathogenesis, and intervention strategies, including vaccine development.

**Results:**

hMPV exhibits global circulation with distinct seasonal and regional patterns. Clinical manifestations often mimic other respiratory viruses, making differential diagnosis challenging. Although no licensed vaccine or specific antiviral treatment currently exists, significant advances have been made in vaccine research, including live‐attenuated, subunit, and mRNA‐based platforms. Public health measures, including hygiene practices, surveillance, and community‐level interventions, have demonstrated effectiveness, particularly in low‐resource settings. Geographic disparities in diagnostic capacity and vaccine research highlight the need for more equitable global responses.

**Conclusion:**

hMPV represents a substantial yet underrecognized burden on global respiratory health. While progress in understanding its molecular biology and vaccine development is encouraging, further research is needed to tailor interventions to diverse populations and the varying capacities of health systems. Strengthened surveillance, global equity in vaccine access, and improved awareness are critical to mitigating the long‐term public health impact of hMPV. The recent outbreaks of hMPV in China stress the urgency of these measures and the need to prioritize the development of effective vaccines and antiviral therapies.

## 1 Introduction

Human metapneumovirus (hMPV) is a negative‐sense, single‐stranded RNA respiratory virus belonging to the family *Pneumoviridae* and the genus *Metapneumovirus*. It primarily affects humans, with infants and young children, older adults, and individuals with weakened immune systems being particularly vulnerable. Infection with hMPV can cause a spectrum of respiratory illness, ranging from mild upper respiratory symptoms similar to the common cold to more severe lower respiratory tract diseases such as bronchiolitis and pneumonia. Since its discovery in 2001, hMPV has been associated with a range of respiratory illnesses, from mild infections to severe pneumonia [1], often co‐circulating with viruses such as respiratory syncytial virus (RSV) and rhinoviruses [2, 3, 4]. Epidemiological studies highlight significant regional variations in hMPV prevalence, with high detection rates in pediatric acute respiratory infections [4, 5]. Seasonal peaks in winter and early spring complicate diagnosis and management, as outbreaks often occur concurrently with other respiratory viruses [6]. Notably, outbreaks in communal settings, such as daycare centers, have demonstrated the virus's potential severity, including fatal outcomes [7].

Clinically, hMPV manifests as bronchiolitis in children and exacerbates conditions such as COPD in adults, particularly those with comorbidities [8, 9]. Coinfections with RSV have been linked to increased disease severity, emphasizing the need for improved diagnostic and therapeutic approaches [8, 10]. Despite its clinical burden, no specific antiviral treatments or vaccines are available, and management remains supportive [11]. Research into host immune responses, particularly interferon‐mediated defense mechanisms, may guide future therapeutic strategies [12, 13]. The economic impact of hMPV is substantial, contributing to increased healthcare costs, hospitalizations, and secondary complications such as acute otitis media [14]. Given its role in respiratory morbidity and mortality, this paper explores the epidemiology, pathogenesis, and public health implications of hMPV.

Despite increasing awareness of hMPV as a significant respiratory pathogen, several critical research gaps persist. For instance, there is currently no licensed vaccine or targeted antiviral treatment, highlighting the urgent need for accelerated vaccine development and therapeutic innovation [15, 16]. Diagnostic limitations, including the sensitivity and specificity of available assays, hinder timely detection and response, particularly in low‐resource settings [17]. The interactions between hMPV and other respiratory viruses, such as RSV and SARS‐CoV‐2, remain underexplored, particularly in terms of their clinical outcomes and immunological mechanisms [10]. Additionally, most epidemiological studies are cross‐sectional and geographically limited, underscoring the need for robust, longitudinal, and multicenter surveillance efforts [18, 19]. Addressing these gaps will be essential to advancing hMPV research and improving global respiratory health.

This paper aims to provide a comprehensive review of the occurrence, epidemiology, and pathogenesis of respiratory infections caused by hMPV, emphasizing its significance as a global public health concern. It critically evaluates current evidence on clinical outcomes, morbidity, mortality, and the burden on the healthcare system, while also assessing existing surveillance, prevention, and intervention strategies. The primary objective is to synthesize existing literature, identify methodological and geographic limitations, and highlight emerging trends and research gaps. By doing so, this review contributes to a more nuanced understanding of hMPV's role in respiratory disease dynamics and provides informed recommendations for future research, diagnostics, and the development of public health policy.

## 2 Methodology

### 2.1 Review Design and Reporting Framework

**Figure 1:**
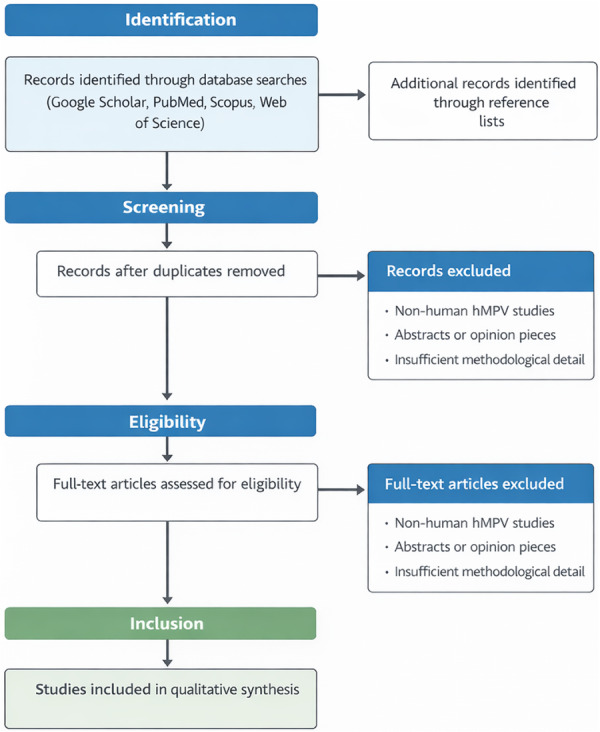
PRISMA‐style flowchart showing the identification, screening, eligibility, and inclusion of studies in this narrative review of human metapneumovirus.

This study was conducted as a structured narrative review and reported using a PRISMA‐informed framework to enhance transparency, reproducibility, and clarity in the processes of literature identification, screening, and synthesis. Although selected elements of the Preferred Reporting Items for Systematic Reviews and Meta‐Analyses (PRISMA) were applied, the review was not designed or conducted as a formal systematic review or meta‐analysis (Figure 1).

### 2.2 Information Sources and Search Strategy

A comprehensive literature search was performed using Google Scholar, PubMed, Scopus, and Web of Science. The search covered publications from January 2001 to March 2025, corresponding to the period following the initial identification of hMPV. To ensure inclusion of recent advances, particular emphasis was placed on studies published between 2015 and 2024. Search terms were used in various combinations and included “human metapneumovirus,” “hMPV,” “respiratory infection,” “epidemiology,” “pathogenesis,” “clinical manifestations,” “diagnosis,” “surveillance,” “vaccine development,” “antiviral therapy,” and “non‐pharmaceutical interventions.” In addition, reference lists of selected articles were manually reviewed to identify further relevant publications.

### 2.3 Eligibility Criteria

Studies were included if they were peer‐reviewed publications written in English and focused on hMPV occurrence, epidemiology, transmission, clinical features, molecular biology, immunopathogenesis, diagnostics, surveillance, public health outcomes, or intervention strategies. Eligible study designs included original research articles, narrative or systematic reviews, surveillance reports, and clinical trial publications. Studies were excluded if they focused exclusively on non‐hMPV species without relevance to human infection, consisted of conference abstracts or opinion pieces lacking substantive data or synthesis, or provided insufficient methodological detail or limited relevance to the objectives of the review.

### 2.4 Study Selection Process

Study selection followed a two‐stage screening process consistent with PRISMA principles. Initially, titles and abstracts retrieved from the database searches were screened for relevance to the review objectives. Subsequently, full‐text articles were assessed for eligibility based on predefined inclusion and exclusion criteria. Screening and eligibility assessments were conducted independently by both authors, and any discrepancies were resolved through discussion and consensus.

### 2.5 Data Extraction

Data were extracted manually using a standardized approach to ensure consistency across studies. Extracted information included publication characteristics and key findings related to hMPV epidemiology, pathogenesis, clinical manifestations, diagnostics, public health outcomes, surveillance approaches, and intervention strategies. No quantitative data pooling or statistical synthesis was undertaken.

### 2.6 Data Synthesis

Given the heterogeneity of study designs, populations, and outcome measures, findings were synthesized using a qualitative narrative approach. Evidence was organized thematically across the major sections of the review, including occurrence and epidemiology, molecular and immunopathogenesis, clinical outcomes, public health outcomes, surveillance and diagnostics, and intervention strategies. Particular emphasis was placed on distinguishing preclinical evidence from early human clinical data, especially in discussions related to vaccine development and the effectiveness of intervention strategies.

### 2.7 Quality and Evidence Considerations

Formal risk‐of‐bias assessment tools were not applied, as the review was not designed as a systematic review. Nevertheless, emphasis was placed on studies with clearly described methodologies, robust data sources, and relevance to clinical and public health practice. Greater weight was given to recent publications and authoritative sources when addressing rapidly evolving topics such as diagnostic innovations, surveillance strategies, and vaccine development.

### 2.8 Reporting and Transparency

This review adhered to PRISMA‐informed reporting principles by clearly documenting the literature search strategy, eligibility criteria, study selection process, and methods of evidence synthesis. No new data were generated, and all conclusions presented are based solely on previously published studies.

## 3 Occurrence and Epidemiology of Human Metapneumovirus

**Figure 2:**
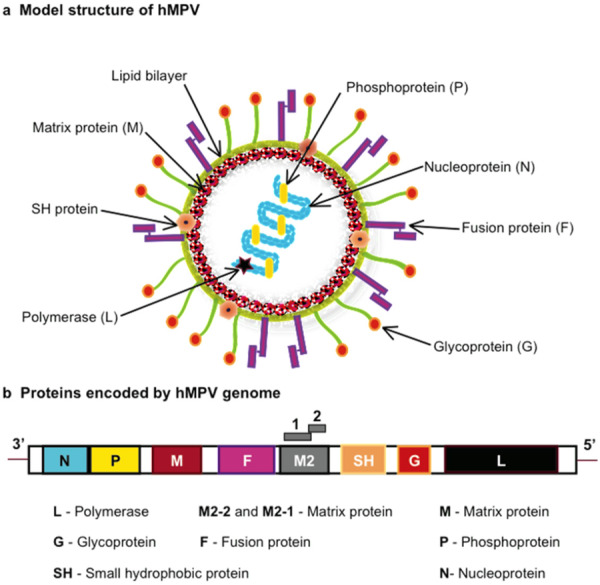
Human metapneumovirus model structure and viral protein (a) and the viral genome (b). *Source:* Cheemarla and Guerrero‐Plata [22].

Human orthopneumovirus is a globally significant respiratory pathogen, especially affecting children, the elderly, and immunocompromised individuals [20, 21]. As a negative‐sense RNA virus from the Pneumoviridae family, hMPV's structural proteins—particularly F and G—facilitate entry, while L, N, M, and M2 support replication and assembly (Figure 2). Although molecular studies have advanced our understanding, much of the evidence is derived from in vitro or animal models, limiting its direct applicability to human populations and vaccine development.

Epidemiological trends vary widely by geography, climate, and diagnostic capacity. Urban areas often report higher prevalence [23], though this may reflect surveillance bias. Seasonality is evident in temperate climates and more diffuse in tropical regions [24, 25], but inconsistent study designs hinder cross‐regional comparisons. Socioeconomic disparities exacerbate underreporting in low‐resource settings due to limited testing infrastructure [19, 26].

Children are consistently identified as the most vulnerable group, but most studies focus on hospitalized cohorts, potentially overestimating disease severity while overlooking community transmission [19]. Studies from Ecuador and Bangladesh confirm hMPV as a significant cause of pediatric pneumonia [27, 28], though small sample sizes and localized contexts limit generalizability. Coinfections with RSV and influenza are common, further complicating attribution of symptoms [14, 29]. High‐risk adults with chronic illnesses face worse outcomes [24, 30], yet these populations remain underrepresented in epidemiological studies. Transmission via droplets, surfaces, and asymptomatic carriers is established [31], though quantifying their relative contributions remains methodologically challenging. The COVID‐19 pandemic disrupted hMPV circulation, revealing shifts in incidence and age patterns following changes in public health behavior [32, 33]. Comparative studies across geographic regions reveal notable discrepancies in the detection and reporting of hMPV. For instance, high‐income countries, such as the United States and Canada, report more consistent seasonal trends, typically peaking in late winter and spring, due to well‐established surveillance networks and molecular diagnostic capabilities [34]. In contrast, low‐ and middle‐income countries (LMICs), such as Bangladesh, Ecuador, and sub‐Saharan African nations, often experience underreporting due to limited diagnostic infrastructure, resulting in incomplete or delayed epidemiological data [26, 27, 28].

Additionally, while temperate regions exhibit well‐defined seasonal outbreaks, tropical settings report year‐round circulation, often coinciding with rainy seasons. This variation highlights the importance of regionally tailored surveillance systems and climate‐sensitive intervention planning [24, 25]. Urban‐rural comparisons also highlight disparities: the higher prevalence in urban centers may reflect both greater transmission potential due to population density and better access to testing, rather than actual differences in disease burden [23].

## 4 Pathogenesis of Human Metapneumovirus

The pathogenesis of hMPV involves a coordinated interplay between viral replication strategies and host immune responses. As a negative‐sense RNA virus, hMPV uses structural proteins—F, G, and N—to mediate attachment, membrane fusion, and replication via RNA‐dependent RNA polymerase [35, 36]. These findings, mainly derived from in vitro and animal studies, provide detailed mechanistic insights but may not fully replicate the complexity of human pathophysiology, particularly across age groups or immunocompromised hosts. hMPV targets respiratory epithelial cells and evades immune detection by inhibiting interferon signaling through SH and M2‐2 proteins [37]. Innate immunity is triggered via pattern recognition receptors (TLRs, RIG‐I), while NK cells and adaptive T‐cell responses are activated. However, a skewed Th2 response may intensify airway inflammation [38]. Although this immune modulation has been well characterized in animal models, human studies remain limited and often lack longitudinal follow‐up to assess long‐term immune outcomes. Additionally, most clinical studies focus on hospitalized patients, limiting insight into milder or asymptomatic cases that may drive transmission.

Reinfection is common due to short‐lived antibody responses [39]; however, population‐based serological studies are scarce, particularly in low‐income settings, which hinders the accurate estimation of population immunity. Risk factors for severe disease—including age, immunosuppression, asthma, and COPD—are well‐documented [19]. However, many studies disproportionately sample pediatric or elderly hospital cohorts, which restricts the demographic scope and external validity [3, 40].

Strain variability also contributes to clinical outcomes, with subgroup A strains being associated with more severe presentations [41]. Yet, genomic surveillance studies remain geographically concentrated in high‐income countries, which limits our understanding of strain circulation and virulence in low‐resource regions. Environmental and social determinants, such as air pollution, overcrowding, and inadequate healthcare access, are often acknowledged but rarely quantified or controlled for in most published studies, particularly outside urban centers. Table 1 summarizes key environmental and social determinants that exacerbate the hMPV burden.

**Table 1:** Environmental and socioeconomic factors influencing hMPV transmission and severity.

Environmental factors	Exacerbating indices	Impact	References
Climate	Cold and dry seasons	It promotes viral survival and increases transmission due to closer human interactions during colder months.	Shaw and Bach [42]; Burbank [43]
Air quality	High levels of air pollution	Weakens respiratory health, increasing susceptibility to severe infections.	Ratajczak et al. [44]; He et al. [45]
Population density	Urban overcrowding	Facilitates higher transmission rates through close contact.	Haas et al. [46]; Pollett et al. [47]
Healthcare access	Limited access to healthcare facilities and antiviral treatments	A delay in diagnosis and treatment can lead to worse outcomes.	Shafagati, Williams [48]
Income level	Low socioeconomic status	Associated with poor nutrition, substandard living conditions, and higher vulnerability to severe infections.	Thörn et al. [49]; Anderson et al. [50]
Education	Lack of awareness and education	This leads to poor hygiene practices and delayed healthcare‐seeking behavior.	Wang et al. [51]; Silva and Santos [52]
Housing conditions	Overcrowded and poorly ventilated living spaces	Increases the likelihood of viral transmission.	Jefferson et al. [53]
Occupational exposure	Jobs requiring high public interaction (e.g., healthcare workers, teachers)	Greater risk of exposure to infected individuals.	Jefferson et al. [53]
Age demographics	A higher proportion of young children and the elderly in vulnerable communities	Increases the population at risk for severe disease outcomes.	Darniot et al. (2009; Hahn et al. [54]

Severe but rare outcomes, such as encephalitis and systemic inflammation, have been reported [46]. However, these findings are primarily based on case studies or small outbreaks [55, 56], raising questions about their generalizability. More recent outbreak reports [57, 58] highlight the virus's impact in long‐term care settings, yet they lack detailed genomic or immunological profiling of affected populations. These limitations highlight a broader need for integrated studies that combine molecular diagnostics, immunological assays, and diverse demographic sampling.

## 5 Clinical Manifestations of Human Metapneumovirus

**Figure 3:**
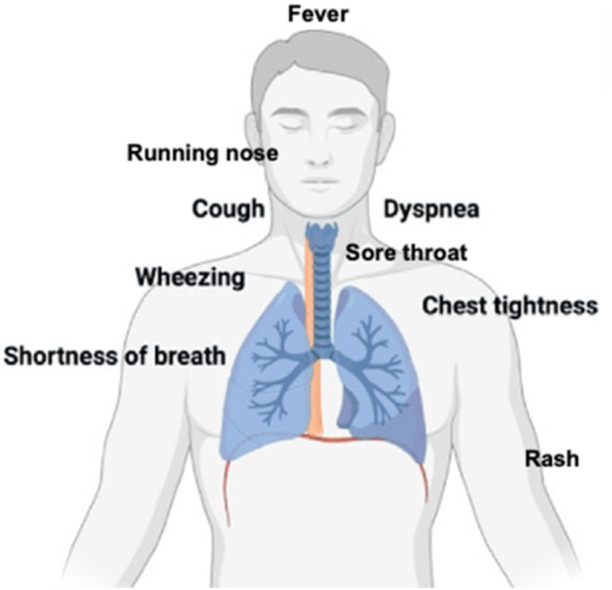
Spectrum of symptoms associated with hMPV.

hMPV causes a spectrum of respiratory illnesses, ranging from mild upper respiratory tract infections to severe lower respiratory diseases, including pneumonia and respiratory failure. Common symptoms include cough, coryza, fever, and wheezing, with severe cases presenting with stridor and hypoxia [59, 60]. Figure 3 shows the common symptoms of Hmpv. The clinical profile of hMPV closely resembles that of RSV, often complicating differential diagnosis [61, 62]. The severity of hMPV infections is influenced by patient demographics and underlying health conditions. Studies indicate that hMPV‐infected children tend to be older than those with RSV, suggesting different susceptibility patterns [63]. hMPV is implicated in 3.8% of acute bronchiolitis cases in infants and has been linked to asthma exacerbations, highlighting its role in chronic respiratory conditions [64, 65].

Both hMPV and RSV exhibit seasonal peaks in winter, but hMPV typically circulates later, from January to April, while RSV peaks from December to February [34, 61]. This overlap can lead to coinfections, though studies suggest that hMPV‐RSV coinfections do not necessarily increase disease severity compared to single‐virus infections [62].

Severe complications are more common in high‐risk groups, such as the elderly and immunocompromised individuals. Lung transplant recipients face a heightened risk, with significant declines in lung function post‐infection [66]. Fatal cases, including hemorrhagic pneumonia in an otherwise healthy child, emphasize the potential severity of hMPV infections [59]. Additionally, hMPV has been associated with long‐term respiratory morbidity in preterm infants, suggesting lasting health consequences [67]. The COVID‐19 pandemic has likely altered hMPV epidemiology, with public health measures impacting respiratory virus circulation. Reports indicate delayed RSV resurgences post‐lockdown, suggesting similar disruptions in hMPV transmission [68]. Understanding these evolving dynamics is essential for public health preparedness and response.

## 6 Public Health Outcomes of Human Metapneumovirus

hMPV is a significant contributor to respiratory illnesses, particularly in young children, the elderly, and immunocompromised individuals. Its burden on healthcare systems is considerable, leading to increased hospitalizations, prolonged stays, and high resource utilization. Studies indicate that preschool children with a history of prematurity face a heightened risk of severe hMPV‐related disease, often requiring intensive medical intervention [69]. The economic impact of these hospitalizations is substantial, with costs comparable to those of influenza and other major respiratory viruses [70]. Morbidity and mortality rates associated with hMPV vary across populations. The annual hospitalization rate for children under five is approximately 1.2 per 1000, comparable to influenza but lower than RSV [50]. Children with pre‐existing conditions are particularly vulnerable, experiencing more severe outcomes and extended hospital stays [71]. Coinfections with RSV and influenza further complicate clinical presentations, increasing disease severity and requiring more comprehensive diagnostic and treatment strategies [72, 73].

The economic burden of hMPV extends beyond direct medical costs, affecting healthcare infrastructure and workforce productivity. A systematic review on respiratory virus‐associated costs highlighted significant financial strain due to hospital admissions, outpatient visits, and lost workdays [74]. Resource‐limited regions are particularly affected, where hMPV can overwhelm already strained healthcare systems [75]. The post‐COVID‐19 surge in pediatric hMPV hospitalizations in Western Australia exemplifies how relaxed public health measures can lead to an increase in respiratory infections, underscoring the importance of long‐term planning for respiratory virus outbreaks [75].

hMPV frequently co‐circulates with other respiratory viruses, with coinfection rates as high as 35% in specific populations [72, 76]. This complicates disease management, as co‐infected patients often experience heightened morbidity and more severe clinical outcomes [41, 73]. A study in Beijing found that hMPV was frequently co‐detected with RSV in children hospitalized with acute respiratory infections, leading to increased hospitalization rates and worsened symptoms [19]. Such findings highlight the need for enhanced public health surveillance and integrated response strategies. The resurgence of hMPV cases post‐COVID‐19 restrictions has drawn renewed attention to its epidemiology. In Western Australia, an increase in pediatric hMPV hospitalizations was observed in a cohort of RSV‐naïve children, highlighting how shifts in public health measures can influence respiratory virus transmission dynamics [75]. Given the seasonal and geographic variability of hMPV, continuous monitoring and targeted healthcare planning are essential for mitigating its public health impact [26].

### 6.1 Surveillance and Diagnostics of Human Metapneumovirus

Effective surveillance and accurate diagnostics are essential for controlling hMPV infections, informing public health responses, and improving clinical outcomes. Surveillance systems enable the assessment of seasonal circulation, geographic spread, and outbreak dynamics, particularly among high‐risk populations such as infants, older adults, and immunocompromised individuals [34, 70]. Current surveillance relies largely on laboratory‐confirmed case reporting supplemented by syndromic surveillance to identify increases in respiratory illness suggestive of outbreaks [77, 78]. However, the absence of routine hMPV testing in many healthcare settings and the resulting underreporting substantially limit accurate burden estimation and global tracking [34, 79].

To overcome these limitations, innovative surveillance approaches—including wastewater‐based epidemiology, artificial intelligence–driven predictive modeling, and genomic surveillance—are increasingly being incorporated into respiratory virus monitoring frameworks [78, 80, 81]. These methods enhance early outbreak detection and improve understanding of transmission dynamics and viral evolution, although their adoption remains uneven due to resource and infrastructure constraints.

### 6.2 Diagnostic Modalities and Use—Case‐Driven Selection

Reverse transcription polymerase chain reaction (RT‐PCR) remains the diagnostic gold standard for hMPV due to its high sensitivity and specificity across clinical settings [82, 83]. Quantitative RT‐PCR additionally allows viral load estimation, which may support clinical assessment and infection prevention measures. Rapid antigen detection tests (RADTs) provide point‐of‐care results and facilitate timely clinical decisions but exhibit lower sensitivity than RT‐PCR, particularly in asymptomatic individuals or later stages of infection [84, 85]. Serological assays, including enzyme‐linked immunosorbent assays (ELISA), are primarily suited for retrospective epidemiological studies, as delayed antibody responses and cross‐reactivity limit their utility in acute diagnosis [86, 87].

Emerging diagnostic technologies such as next‐generation sequencing (NGS) and CRISPR‐based assays offer complementary capabilities. NGS enables detailed strain characterization and outbreak investigation, while CRISPR‐based platforms provide near–RT‐PCR–level sensitivity with simplified workflows [78, 80, 81]. Despite their promise, widespread clinical implementation is constrained by cost, infrastructure requirements, and regulatory considerations.

Because diagnostic objectives differ between acute clinical care and population‐level surveillance, test selection should be guided by context. Table 2 summarizes recommended diagnostic strategies for common clinical and public‐health use cases relevant to hMPV.

**Table 2:** Diagnostic decision aid for hMPV by use case.

Use case	Primary objective	Preferred test(s)	Key considerations
ED triage (symptomatic patients)	Rapid cohorting and clinical decisions	RADT → RT‐PCR if negative with high suspicion	Speed prioritized; confirm negatives in high‐risk cases
NICU/immunocompromised units	Maximize sensitivity and outbreak control	RT‐PCR ± NGS	Lowest limit of detection required; NGS supports transmission analysis
Long‐term care facility surveillance	Early outbreak detection and repeated screening	RT‐PCR (pooled where validated) or high‐performance RADT	Serial testing improves RADT yield
Low‐resource outpatient settings	Accessibility and rapid diagnosis	RADT or CRISPR‐based assays	Minimal infrastructure; CRISPR offers near‐PCR sensitivity
Public‐health surveillance	Trend and variant monitoring	RT‐PCR (sentinel) + NGS	PCR for case detection; NGS for lineage tracking

### 6.3 Diagnostic Performance Characteristics

Reported diagnostic performance varies by assay design, specimen type, timing of collection, and adherence to manufacturer instructions. RT‐PCR assays consistently demonstrate the highest sensitivity and specificity for hMPV detection, whereas RADTs show reduced sensitivity but retain high specificity, particularly during early symptomatic infection. Serial testing strategies can partially compensate for lower sensitivity in rapid assays when used for surveillance or screening. Table 3 summarizes typical performance ranges reported across diagnostic modalities.

**Table 3:** Typical performance characteristics of hMPV diagnostic modalities.

Modality	Typical sensitivity/Performance	Notes
RT‐PCR	Real‐time RT‐PCR is the most sensitive widely used method for hMPV detection, outperforming culture and serology [88], Thus, sensitivity is up to 95‐99%	Gold standard; dependent on sampling quality and timing
RADTs (Rapid antigen tests)	Moderate sensitivity in limited studies. Clinical lysate assays show ~82.3% sensitivity and ~93.8% specificity vs RT‐PCR for hMPV antigen tests [89]	Best early in illness; serial testing improves detection
ELISA (antigen/antibody)	Antigen/serologic antigen methods are noted to lack the high sensitivity of PCR [90]	Limited utility for acute diagnosis
CRISPR‐based assays	Novel CRISPR‐Cas12a combined assays for hMPV demonstrated > 92% agreement with RT‐PCR [91].	Near‐PCR sensitivity; limited availability
NGS (targeted/metagenomic)	Reviews highlight NGS as capable of broad pathogen detection with high analytical specificity, though clinical sensitivity varies [92], detection can be greater than 95%.	Not suitable for acute diagnosis; critical for surveillance

### 6.4 Multiplex Testing and Implementation Considerations

Incorporating hMPV into multiplex respiratory panels can substantially improve detection, particularly given its nonspecific clinical presentation and frequent co‐infection with other respiratory viruses [93, 94]. At minimum, multiplex assays should include hMPV targets and internal controls, with expanded panels incorporating influenza A/B and RSV in acute care and long‐term care settings. Broader panels may be appropriate for pediatric and immunocompromised populations, although increasing panel size may modestly reduce analytical sensitivity per target.

False‐negative results may occur due to improper sample collection, low viral load, or testing outside optimal diagnostic windows [62, 95]. Accordingly, negative RADT results in high‐risk or high‐prevalence settings should prompt confirmatory RT‐PCR testing.

### 6.5 Strengthening hMpv Surveillance

Improving hMPV surveillance requires expanded diagnostic access, strengthened laboratory infrastructure, and systematic inclusion of hMPV in routine respiratory virus testing algorithms [82, 96]. Table 4 summarizes the principal laboratory and surveillance methods used for the detection, monitoring, and characterization of hMPV, outlining their primary applications, operational advantages, and key limitations. It highlights how molecular diagnostics, antigen‐based tests, culture methods, genomic tools, and surveillance approaches complement one another across clinical diagnosis, outbreak monitoring, and research settings (Table 4). Sentinel surveillance networks and enhanced international data sharing are essential for capturing regional and global transmission dynamics [77, 78]. The resurgence of hMPV in Western Australia following COVID‐19 mitigation measures illustrates the virus's capacity for re‐emergence in populations with waning immunity and altered exposure patterns [78]. This underscores the need for sustained surveillance and adaptable public health strategies to mitigate the burden of hMPV on healthcare systems and vulnerable populations [70, 93].

**Table 4:** Diagnostic and surveillance tools for human metapneumovirus.

Method	Primary application	Advantages	Limitations	References
RT‐PCR	hMPV RNA detection	High sensitivity and specificity	Requires specialized equipment	Maertzdorf et al. [97]
Real‐time RT‐PCR	Viral load quantification	Rapid, quantitative	Higher cost and infrastructure needs	Mackay et al. [98]
RADTs	Point‐of‐care antigen detection	Rapid, easy to use	Lower sensitivity than RT‐PCR	Ebihara et al. [84]; Matsuzaki et al. [89]
ELISA	Serosurveillance	Useful for population studies	Limited acute diagnostic value	Hamelin & Boivin [99]
DFA	Antigen detection	Rapid screening	Lower sensitivity; subjective interpretation	Aslanzadeh et al. [61]; Peaper & Landry [100]
Virus isolation	Research and confirmation	Enables viral characterization	Time‐consuming; biosafety requirements	Abiko et al. [101]
Whole‐genome sequencing	Genomic surveillance	Tracks viral evolution	Costly; bioinformatics expertise required	Tulloch et al. [102]; Wei et al. [103]
Syndromic surveillance	Outbreak detection	Real‐time, low cost	Lacks pathogen specificity	Kahn [34]; Jefferson et al. [53]
Biosensors	Experimental diagnostics	High sensitivity; point‐of‐care potential	Early development stage	Khaksarinejad et al. [104]

## 7 Intervention Strategies for Human Metapneumovirus

hMPV intervention strategies primarily focus on prevention, vaccine development, antiviral therapies, and community health promotion [105, 106]. Hygiene practices, social distancing, and masking can reduce respiratory virus transmission, especially during seasonal peaks [107, 108]. Handwashing and use of sanitizers have been associated with lower infection rates, while distancing measures have been used to limit outbreaks [109]. However, much of the effectiveness evidence for these non‐pharmaceutical interventions (NPIs) is derived from broader respiratory virus control (including influenza and SARS‐CoV‐2) rather than being specific to hMPV; studies during COVID‐19 NPIs showed disruption and delay of hMPV seasonality, suggesting indirect hMPV transmission effects rather than hMPV‐specific intervention trials (Foley et al., 2022)

Despite the absence of an approved vaccine, vaccine development for hMPV remains an active field of research. Multiple platforms are under investigation, including live‐attenuated vaccines (LAVs), subunit vaccines, virus‐like particles (VLPs), recombinant vector vaccines, and mRNA‐based platforms.

Importantly, the evidence base is uneven across platforms: most candidates remain preclinical, while human clinical evaluation has been limited and largely early‐phase. At present, the available literature supports early human clinical evaluation for select live‐attenuated designs, whereas subunit, VLP, vector, and mRNA candidates are predominantly supported by animal and other preclinical datasets, and clear documentation of Phase 2 completion for any hMPV monovalent vaccine has not been established [110].

LAVs mimic natural infection and can elicit robust mucosal and systemic immune responses. Promising candidates have demonstrated safety and immunogenicity in animal models and early human evaluation [111, 112]. One live‐attenuated hMPV vaccine candidate has been evaluated in a Phase I clinical trial showing permissive replication and safety signals across adult and seropositive pediatric cohorts, though overattenuation limited replication in seronegative children, and further clinical progression (e.g., Phase II) has yet to be reported [110]. Stability and reversion risks remain key concerns for pediatric use.

- Subunit vaccines use purified viral proteins such as the fusion (F) or glycoprotein (G), often formulated with adjuvants. The F protein, particularly in its pre‐fusion conformation, has emerged as a primary target due to its exposure of neutralizing epitopes [113, 114]. Presently, current clinical registries, most subunit candidates remain in preclinical development or early clinical phase combined RSV/hMPV candidates rather than standalone hMPV Phase II studies. While subunit vaccines are safer, they often require boosters and potent adjuvants to enhance immune responses.
- Virus‐like particles (VLPs) replicate the virus's structure without containing infectious genetic material. VLPs expressing the F protein have induced neutralizing titers in preclinical studies [115]. There are emerging combination candidate programs (e.g., RSV/hMPV mRNA/VLP combinations) progressing into Phase I/II evaluation, but standalone VLP immunogenicity in large human cohorts are rare.
- Recombinant vector vaccines incorporate hMPV genes into viral backbones such as adenoviruses or modified vaccinia Ankara (MVA). These allow heterologous prime‐boost regimens and have shown promise in murine models [116]. ClinicalTrials.gov lists Phase I vectored constructs expressing hMPV elements being tested for safety and immunogenicity in children, representing early human clinical activity distinct from preclinical data [117].
- mRNA vaccines, following the success of COVID‐19 platforms, are being explored for hMPV. mRNA encoding the pre‐fusion F protein is under preclinical evaluation, with potential for rapid deployment and scalable manufacturing [118]. A combined hMPV/RSV mRNA vaccine candidate is currently registered in Phase 1/2 clinical trials to evaluate safety and immunogenicity in adults aged ≥ 60, reinforcing that mRNA hMPV candidates have reached early human study stages, even if Phase II efficacy outcomes are pending [106].

**Figure 4:**
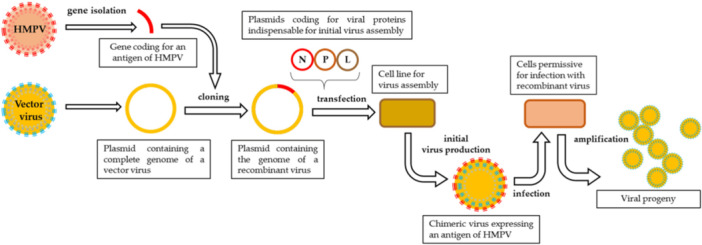
Integration of genetic engineering and virology techniques to generate a functional recombinant virus for studying hMPV and vaccine development. *Source:* Ogonczyk et al. [119].

Using genetic engineering, researchers generate recombinant viruses to study hMPV and develop vaccine candidates (Figure 4) [119]. This process includes: gene isolation and cloning into a vector virus, plasmid transfection into cell lines, and amplification/harvesting of recombinant viral progeny for downstream studies. Parallel to vaccine development, updated intervention strategies increasingly emphasize early detection, particularly in high‐risk settings such as long‐term care homes and pediatric wards. Molecular diagnostic tools (e.g., RT‐PCR multiplex panels) enable faster identification of hMPV, improving patient management and outbreak control [120]. Wastewater surveillance is also being piloted in some countries to track hMPV circulation, building on lessons learned from COVID‐19.

Efforts to tailor strategies to socio‐economic contexts remain essential. In LMICs, where advanced diagnostics may be unavailable, community‐based interventions such as improved ventilation, education campaigns on respiratory hygiene, and mobile health (mHealth) alerts during seasonal peaks may support risk reduction. In contrast, high‐income countries more commonly implement institutional screening, isolation, and infection control protocols—particularly in hospitals and long‐term care settings—supported by stronger laboratory capacity and digital surveillance systems [55, 56].

Public health campaigns promoting hygiene and symptom recognition enhance community resilience [121, 122]. Wastewater monitoring may support early indication of circulation and timely intervention [105]. Engagement through awareness programs can improve adherence to preventive practices [123, 124]. These multifaceted strategies remain central to reducing hMPV burden, with framed emphasis that vaccine claims distinguish early human clinical data from predominantly preclinical evidence, and that NPI effectiveness for hMPV is inferential from broader respiratory virus transmission reduction rather than directly proven in hMPV‐specific controlled trials [78].

Comparison of intervention strategies across regions reveals uneven implementation and effectiveness. High‐income countries generally employ broader testing protocols, routine respiratory panel screening, and institutional infection control policies, particularly in hospitals and long‐term care facilities, supporting earlier detection and containment [55, 56]. In LMICs, reliance on syndromic surveillance and clinical judgment in the absence of molecular confirmation may delay identification and limit timely response [26]. Community‐based interventions and NPIs (e.g., hand hygiene, masking) have been used to reduce transmission during peak seasons and broader lockdown periods [68]. Nevertheless, the evidence for these measures is stronger when linked to general respiratory transmission reduction than when attributed to hMPV‐specific study outcomes [78]. Moreover, while no licensed vaccine exists for hMPV, vaccine research has been concentrated in high‐resource settings, highlighting the need for global equity in preventive innovation. Future strategies should prioritize scalable diagnostics, equitable vaccine access once candidates mature, and climate‐adaptive intervention frameworks.

## 8 Future Directions and Research Needs for Human Metapneumovirus

hMPV remains a significant respiratory pathogen, particularly for children, older people, and immunocompromised individuals. Recent trends in hMPV research have emphasized the need for advanced diagnostic technologies, effective antiviral therapies, and potential vaccines, while highlighting areas that require further exploration. Emerging trends indicate a growing recognition of hMPV's public health implications. Epidemiological studies show that hMPV predominantly affects young children, the elderly, and immunocompromised individuals, underscoring its role as a cause of respiratory illnesses [125, 126]. Moreover, there is an increasing diversification of hMPV strains, with the identification of new subtypes such as A2c and A2b1, which complicate the understanding of its transmission dynamics and pathogenicity [127, 128]. This genetic variability also suggests that vaccine and treatment effectiveness may vary across different populations [28]. Current research gaps are evident, particularly in the development of vaccines. Currently, there are no licensed vaccines or specific antiviral therapies for hMPV despite ongoing efforts [22, 129]. While promising candidates, including those based on mRNA technology and live attenuated viruses, have been developed and even reached early clinical trials [119, 125, 130], challenges remain in ensuring their efficacy across diverse serotypes and understanding host immune responses. Notably, research has pointed out the inadequate exploration of human CD4 T‐cell epitopes, which are crucial for effective vaccine design [130, 131]. Therefore, addressing these gaps in T‐cell immune response could significantly enhance future vaccine strategies. Moreover, research into therapeutic interventions remains limited. Although molecular diagnostics have improved the detection capabilities of hMPV, therapeutic options primarily rely on supportive care [132, 133]. Investigations into synthetic antiviral compounds and monoclonal antibodies are ongoing but have not yet translated into approved therapeutic strategies [134, 135]. New approaches leveraging natural compounds and novel technologies continue to show promise, suggesting future directions for research in this area [134, 136].

Future research should focus on advancing diagnostics, developing antiviral therapies and vaccines, enhancing epidemiological surveillance, and understanding environmental influences on transmission. Current diagnostic tools lack sensitivity and specificity, particularly in high‐risk settings such as long‐term care facilities [28, 137]. While rapid antigen tests show promise, further validation is needed across diverse clinical settings [137]. No approved vaccines or targeted antiviral therapies exist for hMPV, despite its association with severe respiratory illness [138]. Research should explore molecular immunology and mechanisms of pathogenesis [34] to inform therapeutic and vaccine strategies. Data on hMPV prevalence remain fragmented, which limits our understanding of its global burden [139]. Integrated surveillance systems are essential for tracking coinfections with RSV and influenza, particularly among hospitalized children [140, 141]. Strengthening surveillance will better inform public health policies and vaccination strategies [142]. Environmental factors influence hMPV transmission. Climate change may alter the seasonality of viruses, while extreme weather events can disrupt healthcare systems and increase respiratory infections [143, 144]. Urbanization further facilitates the spread of hMPV in crowded environments [145]. Research should examine these interactions to develop climate‐adaptive public health interventions [146]. Addressing these research gaps is crucial for mitigating the impact of hMPV and enhancing global respiratory health outcomes. The interaction between hMPV and co‐circulating respiratory viruses, especially RSV, influenza, and SARS‐CoV‐2, warrants further investigation to understand synergistic effects on disease severity and transmission dynamics. Additionally, gaps in global surveillance data, particularly from LMICs, limit the comprehensive understanding of hMPV's epidemiology.

## 9 Conclusion

Recent outbreaks of hMPV highlight its growing significance as a public health concern, especially among children, older people, and immunocompromised individuals. Its genetic variability, seasonal circulation, and frequent coinfection with other respiratory viruses complicate diagnosis, treatment, and control strategies. These complexities reinforce the need for sustained research and robust surveillance systems. There is a pressing demand for the development of effective vaccines, targeted antiviral therapies, and broadly neutralizing monoclonal antibodies. Equally critical is the advancement of rapid and sensitive diagnostic tools to improve early detection and case management. Global collaboration—through data sharing, coordinated surveillance, and joint research efforts is vital to deepening our understanding of hMPV transmission dynamics and mitigating its impact on overstretched healthcare systems. Moving forward, a comprehensive and integrated response that includes increased research funding, regional preparedness, and public health interventions, such as future vaccination campaigns, will be essential to reducing the disease burden. Strengthening these mechanisms not only addresses hMPV but also builds a proactive framework for tackling similar emerging respiratory pathogens. Ultimately, safeguarding public health in an interconnected world requires collective action, innovation, and sustained commitment.

## Author Contributions

S.C.I. and M.C.O. conceived the review idea, designed the study, and developed the project strategy. S.C.I. and M.C.O. conducted the literature search and synthesis and wrote the original draft of the manuscript. S.C.I. and M.C.O. revised the manuscript for intellectual content. All authors read and approved the final version of the manuscript.

## Funding

The authors received no specific funding for this work.

## Disclosure

The lead author Matthew Chidozie OGWU affirms that this manuscript is an honest, accurate, and transparent account of the study being reported; that no important aspects of the study have been omitted; and that any discrepancies from the study as planned (and, if relevant, registered) have been explained.

## Ethics Statement

The authors have nothing to report.

## Consent

The author expressly consents to the publication of the work.

## Conflicts of Interest

The authors declare no conflicts of interest.

## Data Availability

The authors have nothing to report.
